# Application of a novel anvil holder clamp in laparoscopic gastrectomy

**DOI:** 10.3389/fonc.2025.1557394

**Published:** 2025-04-01

**Authors:** Yuhai Qi, Linchuan Li, Bo Li, Sanyuan Hu, Jiankang Zhu, Guangyong Zhang

**Affiliations:** ^1^ Department of General Surgery, The First Affiliated Hospital of Shandong First Medical University & Shandong Provincial Qianfoshan Hospital, Jinan, Shandong, China; ^2^ Department of General Surgery, Shandong Provincial Qianfoshan Hospital, The First Affiliated Hospital of Shandong First Medical University, Laboratory of Metabolism and Gastrointestinal Tumor, Jinan, Shandong, China

**Keywords:** anastomosis, circular stapler, gastrectomy, laparoscopy, reconstruction methods

## Abstract

**Background:**

The reverse puncture method is a common reconstruction technique used in radical gastrectomy for gastric cancer. However, its widespread use is limited due to unstable clamping and unstable force directions, which complicate the anastomosis process. Therefore, we aimed to develop an improved clamp and investigated its application during the anastomotic process.

**Methods:**

This retrospective study included 29 patients who underwent laparoscopic-assisted Roux-en-Y total gastrectomy (*n*=16) or laparoscopic-assisted gastric tube proximal gastrectomy (*n*=13), utilizing reverse puncture circular anastomosis techniques during surgery. Intraoperatively, the anterior wall of the esophagus was opened using an anvil holder clamp to position the anvil, and a circular stapler was used to transect the esophagus. Finally, circular anastomosis was completed with assistance from our anvil holder clamp. We assessed the number of clamping attempts, time at each stage, clinical characteristics, and surgical outcomes.

**Results:**

All patients underwent successful laparoscopic radical gastrectomy. The mean number of attempts in the two stages was 1.14 and 1.03. The mean duration for these two procedures was 22.6 s and 27.9 s. The overall incidence of postoperative complications (Clavien–Dindo classification grade ≥II) was 17.2%. Esophagojejunostomy leakage occurred in one case (3.4%). Patients with anastomotic leakage were successfully managed with conservative treatment, with no cases of mortality.

**Conclusion:**

Our improved clamp is simple, safe, and effective for the anastomotic laparoscopic gastrectomy procedure and may benefit its wide application.

## 1 Introduction

According to the World Health Organization, gastric cancer is one of the most common malignancies worldwide, with the fifth highest incidence, equating to more than 1 million people diagnosed each year (1). Surgery is often the first option for patients eligible for radical resection of advanced gastric cancer. Laparoscopic radical gastrectomy (LADG) for gastric cancer has become widely accepted since Kitano et al. first described laparoscopic-assisted distal gastrectomy in 1994 (2). Owing to advances in techniques, laparoscopic gastrectomy, including reconstruction of the upper digestive tract, tends to be performed entirely under laparoscopic view. End-to-end reconstruction using a tubular stapler is a standard anastomosis method.

Anastomosis, including esophago-jejunal end-to-side anastomosis or esophago-gastric anastomosis, is the most important and challenging step that could determine the outcome of the operation, especially in the case of the high position of esophageal cutting margin (3). For anastomosis, stabilizing the clamp holder and completing the connection is key to successfully completing digestive tract reconstruction (4). Under normal circumstances, instruments, such as bending separation forceps, are used to clamp the anvil holder, which often has problems such as unstable clamping and uneven force direction, causing difficulties in the anastomosis and even leading to failure or unsatisfactory anastomosis. Therefore, a convenient, fast, and stable device for holding the anvil of a circular stapler is required.

We developed a novel intracorporeal mechanical technique and applied it to the anastomosis stage during laparoscopic gastrectomy. This novel anvil holder clamp, named the “Hug Clamp” (Chinese patent No. 202222089558.4), was designed to be inclined, conforming to the shape direction of the anvil holder. It has the advantages of a strong clamping force and convenient operation. This retrospective observational study aimed to explore the feasibility and effectiveness of our novel anastomosis technique to shorten the anastomosis time, reduce the difficulty of anastomosis, and ensure safety and smooth reconstruction of the digestive tract of patients.

## 2 Materials and methods

### 2.1 Patients

From August 2023 to June 2024, we retrospectively collected the clinical information of 29 patients who were diagnosed with gastric cancer and underwent laparoscopic gastrectomy. Clinicopathological features and surgical outcomes, including demographic data, intraoperative findings, pathological reports, and postoperative recovery details, were recorded. All patients had histologically confirmed gastric adenocarcinomas preoperatively. Inclusion criteria: 1. All patients underwent preoperative gastroscopy, and pathological examination confirmed gastric cancer. 2. No preoperative neoadjuvant therapies such as chemotherapy or radiotherapy. 3. No history of abdominal surgery, no distant metastases of gastric cancer. 4. No contraindications for surgery were found during preoperative examinations. Exclusion criteria: 1. Poor general condition before surgery, unable to tolerate the operation. 2. Preoperative auxiliary examinations showing large tumors, adjacent organ invasion, or distant metastasis. 3. Conversion to open surgery during the procedure.

All procedures were established in accordance with relevant laws and institutional guidelines, and this study received institutional approval. This study was conducted in accordance with the World Medical Association Declaration of Helsinki. Written informed consent was obtained from all patients prior to surgery.

### 2.2 Instrument introduction

**Figure 1:**
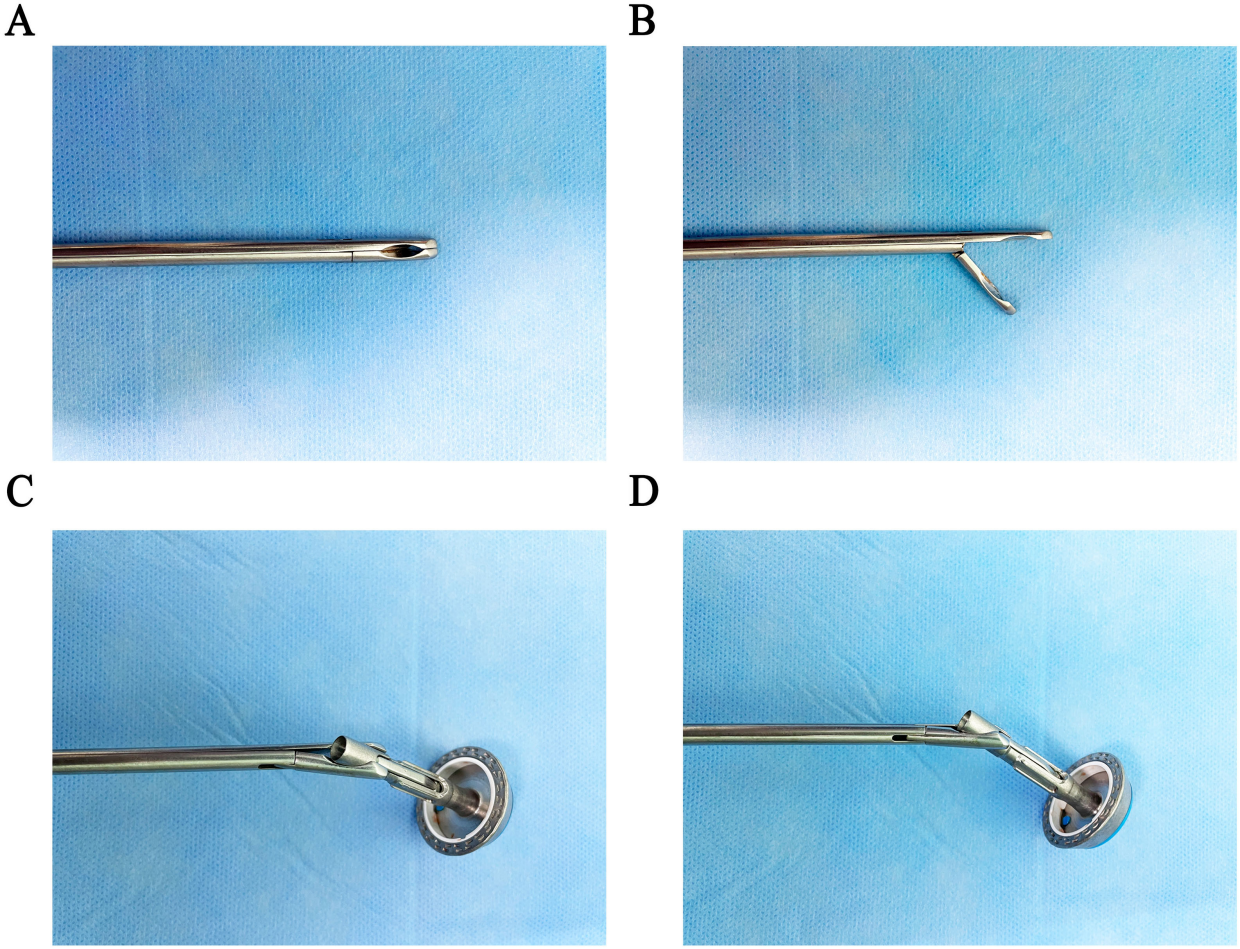
Design of the “Hug Clamp.” **(A)** Closed position of the Hug Clamp. **(B)** Open position of the Hug Clamp. **(C)** Grasping the pin holder in the anterior view. **(D)** Grasping the pin holder in the lateral view.

**Figure 2:**
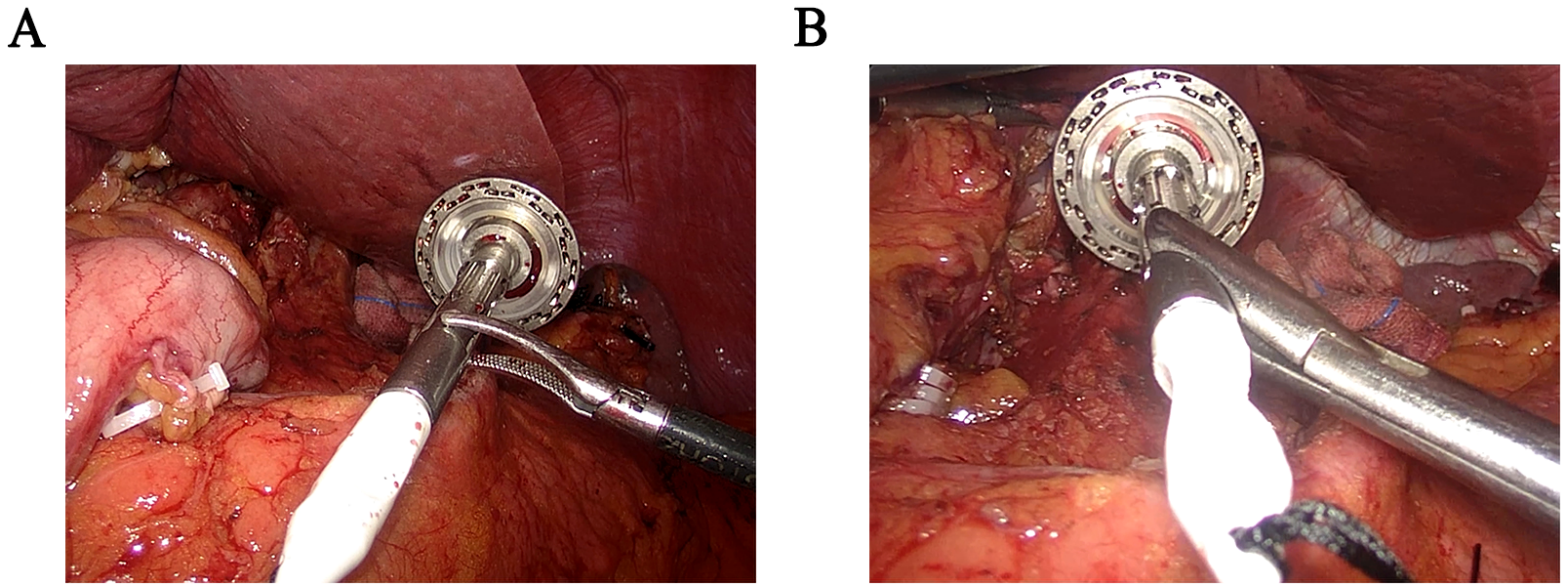
Comparison of intraoperative images between bending separation forceps and “Hug Clamp”. **(A)** Clamp the anvil holder with bending separation forceps. **(B)** Clamp the anvil holder with the “Hug Clamp”.

The anvil holder clamp is a stainless-steel plier body with a head, a rod, and a plier handle. The concave curved surface clamp groove is arranged on the inside of the plier’s head. It is inclined at 30–50 degrees in the direction perpendicular to the opening and closing surfaces of the plier body, which is beneficial for easy, quick, and firm clamping of the circular stapler (**Figure 1**). Compared to the bending separation forceps, our modified holding clamp can clamp the anvil holder more tightly and better accommodate the angle of the esophagus during placement (**Figure 2**). The number of attempts for holding was defined as the time our equipment took to grasp the anvil holder. In contrast, the attempt times for anastomosis were defined as the time our equipment took to grasp the anvil holder and complete the connection with the circular stapler. The duration of clamp holder placement at the end of the esophagus and the duration of holding the anvil holder to complete the anastomosis were also recorded.

### 2.3 Surgical techniques

**Figure 3:**
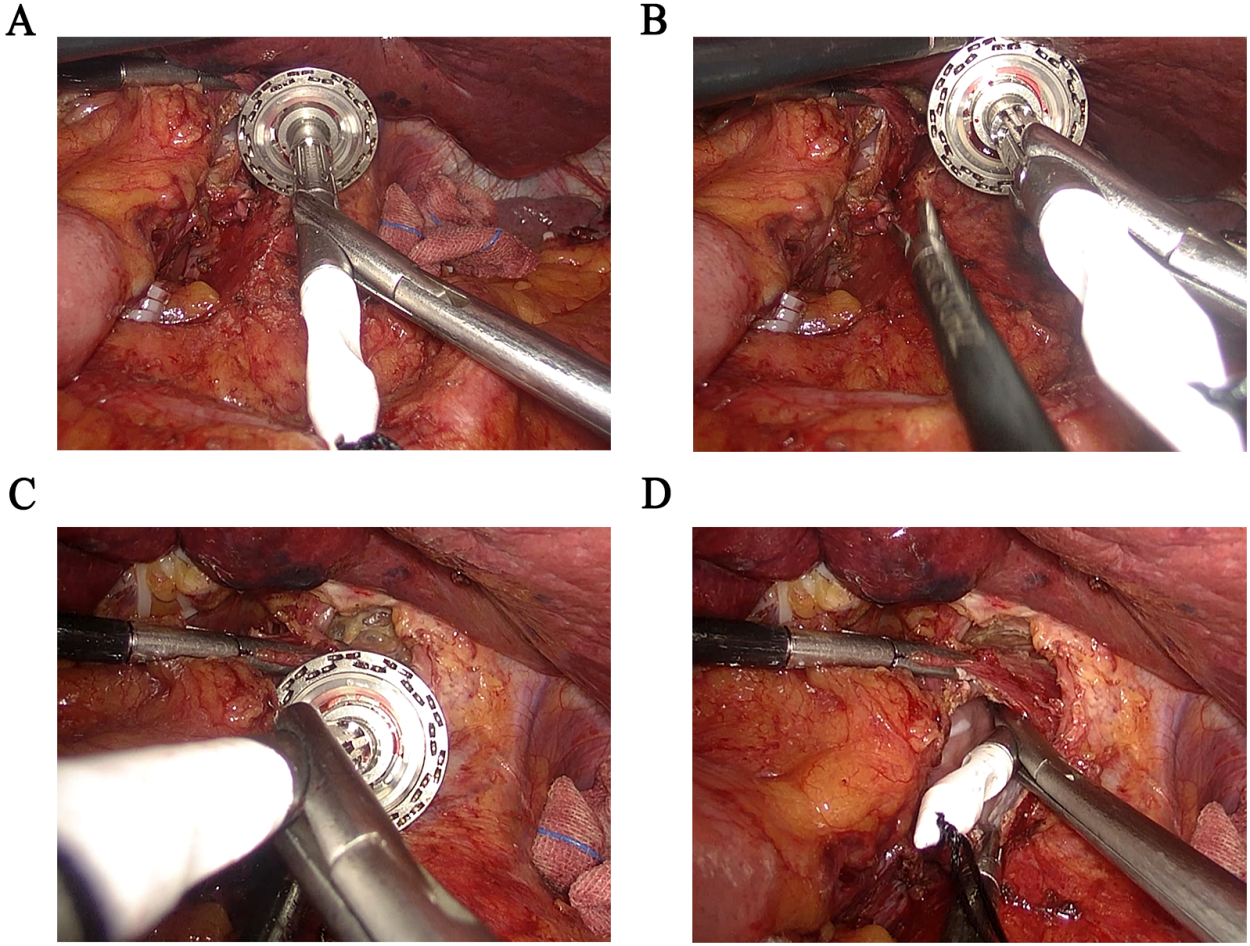
Application of the “Hug Clamp” during laparoscopic gastrectomy. **(A)** Grasping the pin holder. **(B)** Properly expose the cutting end of the esophagus. **(C)** Adjusting the angle properly and putting the pin holder into the cutting end of the esophagus at a certain angle. **(D)** Complete the placement of the anvil holder.

Under general anesthesia, resection and LN dissection were performed with extended D2 + lymphadenectomy based on the Japanese gastric cancer treatment guidelines (5). Per our previous surgical experience, we included dissection of the LNs at stations 1~7, 8a, 9, 11p, 11d and 12a for total gastrectomy. Anastomosis was performed using the circular stapler method as follows. Briefly, an upper midline incision of approximately 5 cm was made through the layers of the abdomen. The anvil stapler was placed in the abdominal cavity, and the pneumoperitoneum was re-established. The front wall of the esophagus was opened under laparoscopy using our anvil holder clamp to put the anvil into the esophagus (**Figure 3**). A linear stapler was used to transect the esophagus. Using the linear stapler, we then performed a side-to-side anastomosis of the proximal and distal jejunum approximately 40 cm from the transected distal end. We sutured continuously to close the shared opening and mesenteric defects. An anastomosis device was inserted into the distal jejunum, the pneumoperitoneum was re-established, and the anvil was pulled out of the esophagus. Anastomosis of esophagojejunostomy was completed with the anvil and stapler using our instruments.

For proximal gastrectomy, based on the Japanese gastric cancer treatment guidelines (5). and our previous surgical experience, we included dissection of the LNs at stations 1, 2, 3a, 4sa, 4sb, 7, 8a, 9, 11p and 11d. The anastomosis was performed using the circular stapler method as follows. An upper abdominal midline incision of approximately 5 cm was made. The anvil stapler was placed into the abdominal cavity, and the pneumoperitoneum was re-established. The anterior wall of the esophagus was opened, the anvil was placed using our anvil holder clamp, and the esophagus was cut with a cutting stapler. The greater omentum and stomach were extracted from the abdominal cavity, and the proximal stomach was severed with a cutting stapler to form a “tube stomach.” After placing an anastomosis device in the remnant, we re-established the pneumoperitoneum. The anvil was extracted from the esophagus, and we performed end-to-side anastomosis between the esophagus and the posterior gastric wall. The gastric remnant was then severed and sealed with a cutting stapler. Subsequently, the anastomotic process was completed.

### 2.4 Postoperative care protocol

Postoperatively, all patients received intravenous antibiotic prophylaxis. The gastric drainage tube was removed without bleeding on postoperative day 1. When tolerable, water sips were permitted from postoperative day 2. The patient was administered a liquid and semifluid diet on postoperative days 5 and 6, respectively. The patient was routinely discharged on day 8 with no postoperative abdominal results. If there were any variations in postoperative recovery, the doctors were permitted to change the treatment strategy to meet the needs of specific situations.

### 2.5 Statistical analysis

Statistical analysis was performed using SPSS 26.0 software. Measurement data following a normal distribution were expressed as mean ± standard deviation (X ± SD), and comparisons between groups were performed using the t-test. Categorical data were expressed as frequency and percentage (%), with differences between groups evaluated using the χ^2^ test or Fisher’s exact test. Statistical significance was indicated by *P*-values, with two-sided *P*<0.05 being considered statistically significant.

## 3 Results

### 3.1 Clinicopathological features of the patients

This study enrolled 29 patients. The clinicopathological characteristics of the patients are shown in **Table 1**. All the patients underwent gastroscopy before surgery and were pathologically diagnosed with gastric cancer. None of the patients had a history of abdominal surgery, and their basic characteristics and clinical information, especially the gastric cancer stage, were recorded. All 29 patients underwent radical gastrectomy, 16 of whom underwent laparoscopic total gastrectomy with Roux-en-Y reconstruction, and 13 underwent laparoscopic proximal gastrectomy with gastric tube reconstruction.

**Table 1:** Characteristics of the patients and tumors.

Characteristics	Total cases (%)/mean ± SD (range)	LATG (Roux-en-Y) Cases (%)/mean ± SD (range)	LAPG (gastric tube) Cases (%)/mean ± SD (range)
Gender			
Male	23 (79.3%)	13 (81.3%)	10 (76.9%)
Female	6 (20.7%)	3 (18.8%)	3 (23.1%)
Age (years)	68.6 ± 5.9 (59–78)	68.3 ± 6.2 (59–78)	68.9 ± 5.6 (59–75)
BMI (kg/m^2^)	25.3 ± 3.5 (17.3–33.2)	24.9 ± 3.6 (17.3–32.9)	25.7 ± 3.5 (19.5–33.2)
Comorbidity			
None	9 (31.0%)	3 (18.8%)	6 (46.2%)
1	9 (31.0%)	6 (37.5%)	3 (23.1%)
≥2	11 (37.9%)	7 (43.8%)	4 (30.8%)
ASA			
Score II	14 (48.3%)	8 (50.0%)	6 (46.2%)
Score III	15 (51.7%)	8 (50.0%)	7 (53.8%)
pT			
T1	9 (31.0%)	2 (12.5%)	7 (53.8%)
T2	2 (6.9%)	1 (6.3%)	1 (7.7%)
T3	6 (20.7%)	3 (18.8%)	3 (23.1%)
T4	12 (41.4%)	10 (62.5%)	2 (15.4%)
pN			
N0	12 (41.4%)	3 (18.8%)	9 (69.2%)
N1	9 (31.0%)	7 (43.8%)	2 (15.4%)
N2	3 (10.3%)	2 (12.5%)	1 (7.7%)
N3	5 (17.2%)	4 (25.0%)	1 (7.7%)
Stage			
IA	7 (24.1%)	1 (6.3%)	6 (46.2%)
IB	4 (13.8%)	2 (12.5%)	2 (15.4%)
IIA	0	0	0
IIB	6 (20.7%)	3 (18.8%)	3 (23.1%)
IIIA	7 (24.1%)	6 (37.5%)	1 (7.7%)
IIIB	2 (6.9%)	1(6.3%)	1 (7.7%)
IIIC	3 (10.3%)	3 (18.8%)	0
Total	29	16	13

ASA, American society of Anesthesiologists; BMI, body mass index; LAPG, laparoscopy-assisted proximal gastrectomy; LATG, laparoscopy-assisted total gastrectomy; SD, standard deviation.

### 3.2 Surgical outcomes

All patients successfully underwent surgery without intraoperative conversion to open surgery. Intraoperative exploration in all patients showed no distant metastases in the abdominal wall, mesentery or other areas. The average duration of surgery was 297.8 min (range: 187–535 min), with an estimated blood loss of 94.8 mL (range: 50–500 mL) (**Table 2**). The average number of retrieved LNs was 26.4, and the average number of positive LNs was 4.1. The average time required to place the clamp holder at the end of the esophagus was 22.6 s. During anastomosis, 25 patients required modified clamping forceps to clamp the anvil only once (86.2%), while four patients required clamping twice (13.8%). The average anastomosis time (from pulling the anvil from the esophagus to completing the esophago-gastric or esophago-jejunal anastomosis) was 27.9 s. Only one patient underwent two grasps for the anvil; the other 28 required only one grasp. The anastomosis sites in all 29 patients were reinforced with interrupted sutures of absorbable material. None of the patients required the initial incision to be extended intraoperatively because of difficulties with the anastomosis.

**Table 2:** Surgical outcomes.

Outcomes	Total cases (%)/mean ± SD (range)	LATG (Roux-en-Y) Cases (%)/mean ± SD (range)	LAPG (gastric tube) Cases (%)/mean ± SD (range)
Time of operation (min)	297.8 ± 68.9 (187–535)	297.6 ± 52.6 (187–401)	298.2 ± 87.4 (191–535)
Estimated blood loss (mL)	94.8 ± 87.0 (50–500)	106.3 ± 113.8 (50–500)	80.8 ± 32.5 (50–150)
Retrieved lymph nodes	26.4 ± 11.3 (8–60)	29.9 ± 12.2 (16–60)	22.0 ± 8.6 (8–37)
Positive lymph nodes	4.1 ± 8.0 (0–35)	6.5 ± 10.0 (0–35)	1.1 ± 2.4 (0–8)
Times^a^			
Once	25 (86.2%)	12 (75.0%)	13 (100%)
Twice	4 (13.8%)	4 (25.0%)	0
Mean	1.14	1.25	1
Time^b^ (s)	22.6 ± 4.2 (15–32)	21.3 ± 3.3 (17–30)	24.2 ± 4.7 (15–32)
Times^c^			
Once	28 (96.6%)	16 (100%)	12 (92.3%)
Twice	1 (3.4%)	0	1 (7.7%)
Mean	1.03	1	1.08
Time^d^ (s)	27.9 ± 4.2 (21–35)	27.2 ± 3.6 (22–33)	28.7 ± 4.8 (21–35)

SD, standard deviation.

Times^a^ means the times to grasp the anvil holder when putting it in the esophagus.

Time^b^ (s) means the time when the clamp holder was placed into the broken end of the esophagus.

Times^c^ means the times to grasp the anvil holder during the anastomosis.

Time^d^ (s) means the time holding the anvil holder until the completion of the anastomosis.

### 3.3 Clamping, anvil placement, and anastomosis times in patients with different body mass index levels

We documented the number of attempts to grasp the anvil holder during insertion into the esophagus and the anastomosis, the time that the clamp holder was positioned at the broken end of the esophagus, and the moment when the anvil holder was held to complete the anastomosis. We further analyzed the patients per their BMI to explore the efficacy of our equipment in patients with obesity. Patients were divided into two groups (BMI ≥24 kg/m^2^ and BMI <24 kg/m^2^), and the number of clamping attempts and the times for anvil placement and anastomosis for all patients and for LATG (Roux-en-Y) and LAPG (gastric tube) procedures were analyzed. Among the 29 patients, 19 had BMI ≥24 kg/m^2^, whereas 10 had BMI <24 kg/m^2^. In the BMI <24 group, when put the anvil in the esophagus, 8 patients required modified clamping forceps to clamp the anvil holder only once (80.0%), while 2 patients required clamping twice (20.0%). The average placement time (the time when the clamp holder was placed into the broken end of the esophagus) was 22.2 s. When grasp the anvil holder during the anastomosis, 9 patients required modified clamping forceps to clamp the anvil only once (90.0%), while 1 patient required clamping twice (10.0%). The average anastomosis time (the time holding the anvil holder until the completion of the anastomosis) was 28.5 s. In the BMI≥24 group, when put the anvil in the esophagus, 17 patients required modified clamping forceps to clamp the anvil only once (89.5%), while 2 patients required clamping twice (10.5%). The average placement time (the time when the clamp holder was placed into the broken end of the esophagus.) was 22.8 s. When grasp the anvil holder during the anastomosis, all 19 patients required modified clamping forceps to clamp the anvil only once (100.0%). The average anastomosis time (the time holding the anvil holder until the completion of the anastomosis) was 27.5 s. There were no significant differences in the attempt times and duration to grasp the anvil and finish the connections between the different BMI groups (**Table 3**).

**Table 3:** Comparison of different BMI groups.

Characteristic	BMI <24(kg/m^2^) Cases (%)/mean ± SD	BMI ≥24(kg/m^2^) Cases (%)/mean ± SD	*P*
	*N*=10	*N*=19	
Gender			1.000
Male	8 (80.0%)	15 (78.9%)	
Female	2 (20.0%)	4 (21.1%)	
Age (years)	72.1 ± 3.1	66.7 ± 6.2	0.004
Comorbidity			0.884
None	4 (40.0%)	5 (26.3%)	
1	3 (30.0%)	6 (31.6%)	
≥2	3 (30.0%)	8 (42.1%)	
Times^a^			0.592
Once	8 (80.0%)	17 (89.5%)	
Twice	2 (20.0%)	2 (10.5%)	
Time^b^ (s)	22.2 ± 5.1	22.8 ± 3.8	0.726
Times^c^			0.345
Once	9 (90.0%)	19 (100.0%)	
Twice	1 (10.0%)	0 (0.0%)	
Time^d^ (s)	28.5 ± 3.3	27.5 ± 4.6	0.558

BMI, body mass index; SD, standard deviation.

Times^a^ means the times to grasp the anvil holder when putting it in the esophagus.

Time^b^ (s) means the time when the clamp holder was placed into the broken end of the esophagus.

Times^c^ means the times to grasp the anvil holder during the anastomosis.

Time^d^ (s) means the time holding the anvil holder until the completion of the anastomosis.

### 3.4 Postoperative outcomes

The overall postoperative complication rate (grade ≥II) was 17.2%, with esophago-jejunal anastomotic complications occurring in 3.4% of cases, which was postoperative esophago-jejunal anastomotic fistulas. Leading the list of other complications were pneumonia (three patients, 10.3%) and respiratory failure (three patients, 10.3%), followed by heart failure (one patient, 3.4%), bacteremia (one patient, 3.4%). All postoperative complications were successfully treated, with no postoperative deaths (**Table 4**).

**Table 4:** Postoperative outcomes.

Outcomes	Total cases (%)/mean ± SD (range)	LATG (Roux-en-Y) Cases (%)/mean ± SD (range)	LAPG (gastric tube) Cases (%)/mean ± SD (range)
Overall complications*	5 (17.2%)	3 (18.8%)	2 (15.4%)
Grade II	1 (3.4%)	0	1 (7.7%)
Grade III	1 (3.4%)	1 (6.3%)	0
Grade IV	3 (10.3%)	2 (12.5%)	1 (7.7%)
Complications at the esophagojejunostomy site			
Anastomotic leakage	1 (3.4%)	1 (6.3%)	0
Anastomotic bleeding	0	0	0
Anastomotic stenosis	0	0	0
Hospitalization cost(¥)	65811.5 ± 9673.0(50017-89878)	66067.9 ± 10482.9(51367-89878)	65495.9 ± 8986.9(50017-85191)
Hospital stay (days)	21.2 ± 10.9 (11–60)	23.9 ± 13.9 (11–60)	17.9 ± 4.2 (12–25)
Postoperative hospital stay (days)	13.6 ± 7.7 (9–45)	15.3 ± 9.8 (9–45)	11.5 ± 2.9 (9–19)
Mortality	0	0	0

SD, standard deviation.

*Complications grade II or higher according to the Clavien–Dindo classification.

## 4 Discussion

Our study reports novel laparoscopic equipment that can be used in the anastomotic process of laparoscopic gastrectomy, mainly in the reverse puncture procedure in LATG and gastric tube reconstruction in LAPG. As far as we know, this is the first report on the modification of the anvil clamping device, verifying its safety and efficacy in anastomotic procedures.

Over the past decade, although the incidence and mortality rates of gastric cancer have declined, its prognosis remains poor, posing a serious threat to human life and health (6). Currently, the incidence of distal gastric cancer is gradually decreasing worldwide, while the incidence of proximal gastric cancer and gastroesophageal junction cancer continues to rise (7). With the rising detection rates of proximal gastric cancer and gastroesophageal junction cancer, total and proximal gastrectomy are increasingly performed, and their safety and efficacy have been confirmed (8–10).

For patients with locally advanced gastric cancer, D2 LN dissection is recommended during radical gastrectomy (11). Following LN and tumor dissection, the most important factor closely related to postoperative recovery is digestive tract reconstruction. Currently, the methods of digestive tract reconstruction in radical gastrectomy are diverse and can be classified into circular stapler anastomosis, linear stapler anastomosis, and Hand-sewn anastomosis based on the anastomotic devices (12). Common methods of circular stapler anastomosis include hand-sewn purse-string sutures, reverse puncture, and OrVil devices (13–15). Common methods for linear cutting stapler anastomosis include the functional end-to-end esophagojejunostomy (FEEA), side-to-side esophagojejunostomy (Overlap), and π-shaped anastomosis (16–18). The circular stapler is most commonly used for anastomosis among these reconstruction methods.

The reverse puncture method, first introduced by Omori et al. in 2009 (19), is widely used for digestive tract reconstruction using circular staplers. This method simplifies the insertion of the anvil into the esophageal stump by avoiding the need for complex purse-string placement, thereby enhancing the efficiency of the esophagojejunostomy. Additionally, this method is beneficial because of its simplicity and reduced risk of infection (20). However, reverse puncture anastomosis can still be optimized further, especially for obese patients where performing the reverse puncture technique under laparoscopy remains challenging. This is due to exposure difficulties caused by excess abdominal fat and the insertion of the anvil holder due to the higher gastroesophageal junction (21). Therefore, recently, researchers have made improvements to circular anastomosis techniques with preliminary success, although a standardized and simplified procedure has not yet been determined (20, 22, 23).

Some researchers have also revealed difficulties in the anastomotic procedure using the reverse puncture method. Kazuhiro et al. showed that three patients experienced anastomotic issues during insertion of the anvil head. Two patients developed postoperative anastomotic leakage, which may be due to tears in the esophagus during insertion. Therefore, a simple and stable method to insert the anvil head easily and quickly is critical for successful completion of the anastomosis (24).

Based on the above problems and practical clinical needs, we invented this novel anvil holder device named the “Hug Clamp.” A concave curved surface clamping groove is arranged on the inside of the plier’s head at an inclined angle perpendicular to the opening and closing surface of the plier’s body. This enabled it to tightly grasp the anvil holder of the circular stapler. Based on the application of the device, the anvil holder of the circular stapler can be clamped conveniently, quickly, and stably. Furthermore, the tilt direction of the anvil holder following clamping is more suitable for surgical operation and can be easily inserted into the esophageal incision, even at a high position for esophageal dissection. As a result, anastomosis after total and proximal gastrectomy is flexible and practical, reducing the operator’s experience requirements and improving the operation’s efficiency.

In this study, we used this novel device in a reverse puncture operation in 29 patients who underwent LATG or LAPG. The surgery was successfully conducted in all patients, all patients recovered after surgery and were discharged. Throughout the anastomotic procedure, we recorded the attempt times to grasp the anvil holder for placement in the esophagus and performing anastomosis. The mean attempt times were 1.14 and 1.03, respectively, indicating that the anvil holder could be grasped on the first attempt and tightly combined during the entire anastomosis process. In addition, we recorded the time at which the clamp holder was placed on the oral side of the dissected esophagus and when the anvil holder was held to complete the anastomosis. The time of these two procedures was 22.6 s and 27.9 s, respectively, which was quickly and easily achieved.

Previous studies have shown that the anvil placement time using the reverse puncture method ranges from 9.0 to 12.6 min, while the duration of exact procedure was not defined clearly (19, 22, 23). In our study, we only recorded the time to grasp the anvil and place it into the esophagus, which was 22.6 s, most of which were grasped firmly and quickly on the first attempt. This suggests that the improved clamp allows for convenient, rapid, and stable gripping of the circular anvil, which was the first report to introduce such improvements to clamp devices.

Previous studies have shown that the incidence of anastomotic leakage after laparoscopic radical gastrectomy ranges from 2.1% to 14.6% (25). In this study, none of the patients experienced anastomotic bleeding or stenosis, and the esophagojejunostomy leakage rate was 3.4%, which is consistent with previous results. Factors influencing anastomotic leakage include tension and blood supply at the anastomosis site, internal stimulation by digestive fluids, and patient factors such as diabetes and nutritional status (24, 26–28). Although we were satisfied with the anastomosis result during surgery, with good tension and blood flow at the anastomotic site, however, one patient who underwent LATG developed anastomotic leak postoperatively. We investigated the cause of anastomotic leak in the patient. Intraoperatively, we found that the tumor was large and at an advanced stage, which located at the esophagogastric junction, and there was significant edema at the junction. The patient was elderly, had poor nutritional status, although adequate postoperative nutritional support was provided, the albumin level remained low. This may have contributed to the anastomotic leak in this patient. The patient recovered after active conservative treatment and was discharged 1 month after surgery.

Due to the limited intra-abdominal working space in obese patients, which often causes operational difficulties. In this study, we analyzed the relationship between attempt times, duration, and BMI to further explore whether our equipment is suitable for obese patients. Among patients with BMI ≥24 kg/m^2^, our results revealed that the attempt times for grasping and anastomosis were 1.11 and 1.00, respectively. Furthermore, the time for clamp holder placement into the end of the esophagus and the time holding the anvil holder to complete anastomosis was 22.8 s and 27.5 s, respectively, which showed no significant differences compared to patients with BMI <24 kg/m^2^. Although there was a significant difference in age between the two groups, according to our previous clinical practice, the difference in age did not have a remarkable impact on the operation of anastomosis. These results indicated that our novel instrument can be safely and successfully used for anastomosis in obese patients, similar to its advantages in normal-weight patients.

Our study has some limitations. We reported the initial results of the application of this novel instrument; however, the number of patients was relatively small, and the instrument was only used in our center. Therefore, the instrument needs to be applied to more patients and medical centers to further determine its therapeutic effects. In addition, for the comparison of patients with different BMI, the differences in age may lead to certain bias of the results. The subsequent study on a larger scale of patients is needed to further confirm our conclusions. Besides, the highest BMI of all patients was 33.2 kg/m^2^, therefore, the effect of our device on severely obese patients requires further investigation.

In conclusion, we invented a novel clamp device to firmly grasp the anvil holder, which is the first report of this type of device. This equipment could hold the anvil holder tightly and could be easily placed at the end of the esophagus at a certain angle during reverse-puncture anastomosis. In our application, the device showed promising effects, which are expected to solve the difficulties encountered in circular anastomosis. Therefore, further studies on this device are warranted.

## Data Availability

The original contributions presented in the study are included in the article/supplementary material. Further inquiries can be directed to the corresponding authors.
